# Applied Improvisation Enhances the Effects of Behavioral Activation on Symptoms of Depression and PTSD in High School Students Affected by the Great East Japan Earthquake

**DOI:** 10.3389/fpsyg.2021.687906

**Published:** 2021-08-26

**Authors:** Chikaze Sugiyama, Shunsuke Koseki, Yoko Niikawa, Daisuke Ito, Fumito Takahashi, Rie Ishikawa

**Affiliations:** ^1^Graduate School of International Studies, J. F. Oberlin University, Machida, Japan; ^2^Faculty of Psychology and Education, J. F. Oberlin University, Machida, Japan; ^3^Graduate School of Psychology, J. F. Oberlin University, Machida, Japan; ^4^Graduate School of Education, Hyogo University of Teacher Education, Kato, Japan; ^5^Institute of Education, Shinshu University, Matsumoto, Japan

**Keywords:** depression, PTSD, school based intervention, behavioral activation, applied improv, students, the Great East Japan Earthquake

## Abstract

**Background:**

The education system can serve as a community-based resource to support the provision of long-term follow-up care after large-scale disasters. While school-based interventions conducted after a disaster have been confirmed to reduce symptoms of depression and posttraumatic stress disorder (PTSD), adolescents often exhibit low treatment motivation. Traditional methods used to encourage treatment motivation include fun activities, such as applied improv (AIM). This study evaluated the intervention effects and improved motivation of an intervention program combining AIM with the behavioral activation approach (BAA).

**Methods:**

Participants were 253 tenth graders, who were in fifth grade at the time the Great East Japan Earthquake of 2011, and 239 students were included in the analyses. Participants were divided into two groups: the BAA and AIM + BAA groups. Students in each group participated in one class-wide intervention session, which lasted 60 min. Depression, PTSD symptoms, behavioral activation, avoidance, and resilience were evaluated using psychological scales. A participant’s evaluations of the intervention were confirmed using the impression sheet consisting of six items that measure comprehension, difficulty, efficacy, generalization, confirmation of a specific situation, and motivation.

**Results:**

A two-way analysis of variance (ANOVA) conducted using data from the psychological scale did not reveal a significant effect from the intervention program. However, the Mann-Whitney *U*-test, which used data from the impression sheet, showed a significant effect on comprehension (*p* = 0.001), generalization (*p* = 0.023), and motivation (*p* = 0.025).

**Conclusion:**

This study did not confirm the effectiveness of the BAA in reducing symptoms of depression and PTSD in adolescents. Regarding treatment motivation, the AIM + BAA group reported higher motivation than the BAA group. Thus, one session of AIM may contribute to improved treatment motivation in adolescents. AIM creates a safe environment and encourages engagement and participation in interventions. Treatment motivation is an important issue in adolescent therapy, and AIM may help solve this problem.

## Introduction

Social concern over mental health problems after a large-scale disaster has increased in recent years. The experience of being affected by a large-scale natural disaster may put one at risk of developing mental health problems, such as depression and posttraumatic stress disorder (PTSD), especially in children and adolescents (Vogel and Vernberg, 1993; Norris et al., 2002; Furr et al., 2010; Tang et al., 2014, 2017). For example, following the Almeria earthquake in 1988, a high incidence of depression and PTSD after 18 months (Goenjian et al., 1994), and a high incidence of chronic depression and severe PTSD symptoms after 4.5 years (Goenjian et al., 2000) were reported. After such a large-scale disaster, psychological treatment centered on symptoms of depression and PTSD is needed. Psychological responses to disasters usually decrease after 18 months to 3 years; however, these issues can become chronic without proper treatment (Vogel and Vernberg, 1993). Moreover, children and adolescents need psychological treatment following a disaster similar to that of adults (Okuyama et al., 2016). Following the Great East Japan Earthquake of 2011, which was the most serious disaster in Japan since World War II, the students affected by this disaster showed a high risk of developing depression and PTSD after 4 years (Koseki et al., 2013; Nakaza et al., 2017). Therefore, psychological treatment for mental health problems following a major disaster should not be limited to immediate intensive interventions post-disaster (Vernberg et al., 2008) but include additional community-based treatment options as well.

With respect to additional treatment options available within the community, school-based intervention has shown to be a good option (Klingman, 1993). Schools are good community resources that can provide long-term follow-up and have the advantage of providing psychosocial treatment post-disaster for all children in the area. Furthermore, schools are a good place to provide safe and supportive interventions following a major disaster. In recent years, models have been developed to provide gradual, beneficial support in schools depending on the support needs of the students (McDermott, 2014). This makes it possible to respond to sudden clinical demands after a disaster or provide medium- to long-term care to students within the community (McDermott and Cobham, 2014). Many researchers have reported improvements in depression (Calear and Christensen, 2010; Stallard and Buck, 2013) and PTSD (Rolfsnes and Idsoe, 2011; Jaycox et al., 2012) following school-based interventions. School-based intervention can consist of many components and methods, including the behavioral activation approach (BAA).

BAA is based on the behavioral activation model (Jacobson et al., 2001), where, activation of behaviors increases opportunities for positive reinforcement and reduces depression condition; conversely, avoidance indicates less chances of reinforcement in decreasing depressive symptoms. According to the behavioral activation model, a common tendency among depressed patients is to engage in avoidance behaviors and lose the opportunity to obtain reinforcement. The reinforcement refers to experiences that are enjoyable. These missed opportunities are thought to increase the persistence of depression. In contrast, the model suggested that increasing activity through BAA can increase the chances of obtaining enjoyable experiences. Notably, a meta-analysis of behavioral activation treatments for depression reported a high effect size (Cuijpers et al., 2007; Mazzucchelli et al., 2009; Sturmey, 2009). BAA improves individual well-being by reducing avoidance (Jacobson et al., 2001; McCauley et al., 2016) and promoting resilience (Reynolds, 2019; Sugiyama et al., 2020). Resilience is defined as the process of, capacity for, or outcome of successful adaptation despite challenging or threatening circumstances (Asukai et al., 2002). Resilience is improved by acquiring methods and resources to effectively cope with various daily stressors. Improving resilience leads to the reduction and prevention of depressive symptoms, functioning as a protective factor against depression (Martell et al., 2001). Therefore, school-based interventions, including BAA, which improve activation, avoidance, and resilience, can be thought to contribute to adolescent mental health.

Interventions for adolescents often face the problem of low treatment motivation (Pekarik and Stephenson, 1988; Hirakawa et al., 2005). One reason for this is that adolescents tend to have a greater fear of negative evaluation from others compared to adults (McClure and Pine, 2006). In adolescence, students can become more reluctant to participate in group interventions, including activities with classmates, which can have a negative impact on intervention effects. For example, some students reported that a class-based intervention using the cognitive reconstruction method was “not fun” (Koseki et al., 2007). Additionally, adolescents may not feel motivated to participate in interventions because they do not feel such programs are necessary; particularly the ones who have not yet developed any mental health problems or symptoms. It has been noted that pre-treatment motivation can influence the effectiveness of subsequent treatments (Westra et al., 2011). Hence, as treatment motivation is important in interventions for adolescents, a method to promote motivation for treatment is also needed.

A traditional method of encouraging participation in children and adolescents is the use of fun activities, such as applied improv (AIM). AIM comes from the field of improvisational drama and was designed as an actor-training method; however, it has come to be used for purposes other than dramatic performances, such as education and corporate training (Holzman, 2016). AIM is based on the principle of “Yes, and.” This principle implies that you should accept whatever the other person says without denying it (Yes) and develop the story by adding your own ideas (and) (Johnstone, 2014). This principle is said to create the receptive atmosphere necessary to motivate treatment. Previous studies reported improvements in depression and anxiety in adolescents and adults (Felsman et al., 2019) and a reduction in aggressive behavior in children with the use of AIM (Kisiel et al., 2006). These fun activities may improve treatment motivation; however, symptom reduction after previous treatment proceeds may also increase motivation. To our knowledge, there is currently no research yielding suggestions for increasing motivation at the onset or early stages of treatment in school-based interventions using such motivation-enhancing procedures.

The purpose of this study was to evaluate the effects of BAA and AIM on symptoms of depression and PTSD symptoms in adolescents as the primary outcome measure. This study also explored improvements in the secondary outcome measures of behavioral activation, avoidance, and resilience, which constitute a possible intervention mechanism of BAA. In addition, the study explored whether intervention motivation could be increased through the use AIM.

## Materials and Methods

### Participants

Participants were 253 tenth graders selected using convenience sampling. Mean participant age was 15.17 years (*SD* = 0.37), and there were 73 boys and 180 girls. The participating high school was located in Morioka city, where more than 290,000 citizens suffered from the Great East Japan Earthquake of 2011. The students who participated in the present study were in the fifth grade of elementary school at the time of the disaster.

The first questionnaire survey was conducted in May 2018 pre-intervention. The intervention was conducted in June 2018. A second questionnaire survey was conducted 2 weeks later. The authors were not present for the questionnaire survey. It was administered in the classroom by the homeroom teacher. Participants were assigned to either the BAA group or AIM + BAA group according to their homeroom class, with 108 (three classes) and 145 students (four classes) in the BAA and AIM + BAA groups, respectively. Students in both the groups participated in one class-wide intervention session, which lasted 60 min.

All participating students, their parents or guardians, and the school principal provided informed consent prior to completing the first questionnaire. The questionnaire was anonymous and did not include any personal information. The study protocol was approved by the ethics committee of the first author’s institution. No participants had received psychotherapy or pharmacotherapy prior to this study.

### Intervention Program for the AIM + BAA Group

The AIM portion of the intervention was conducted by a graduate student majoring in clinical psychology, while the BAA portion was conducted by a university teacher who specializes in cognitive behavioral therapy. The intervention took place in the high school gymnasium. At the beginning of the AIM + BAA intervention (Table 1) (first component), participants were provided with explanations about the program proceedings (approximately 2 min) and AIM (approximately 3 min).

**TABLE 1:** Summary of the intervention program for AIM + BAA group.

	Component	Exercise
1	Introduction	The explanation on how to proceed with the program.
2	AIM	Participants play the pantomimed game of catchball.
3	BAA	Participants learn about can be get good results by behavioral activation.
4	BAA (Work)	Participants looked back on their own actions and held discussions and work to think about what kind of actions were beneficial.
5	Conclusion	For the purpose of generalization, I told them that action activation is beneficial in daily life.

*AIM, Applied improv; BAA, Behavioral activation approach.*

During the second component, participants played pantomime game of catch (about 15 min) (Kinugawa, 2002). Participants were divided into small groups of 5-6 students, where each group was asked to form a circle. They were then asked to throw an imaginary object, such as a rubber ball, and continue to cooperate as much as possible. Participants were instructed to make eye contact as much as possible and throw to the other party in an easy-to-understand manner. After three rounds of pantomimed catch, the researcher conducting the intervention thanked the students for their willingness to participate. In addition, we provided feedback emphasizing that communication with others can be promoted by actions such as gesture and eye contact.

In the third and fourth components, participants learned using BAA that they could obtain good reinforcement by behavioral activation (approximately 35 min). The main theme of this component was to “learn about the impressions of actions on others.” Through the use of PowerPoint and role-play, we presented a scene showing how a good impression could be made through one’s actions. In that scene, two boys saw that one of their friends achieved a milestone. One of the boys did not greet the friend because he felt disappointed, jealous, or any other negative emotions, giving a bad impression. The other boy greeted him regardless of his negative emotions, making a good impression. Through this example, the researcher explained that activating a behavior is more important than whether we want to act or not. They also explained that it is easier to get better results when one acts than when one does not. The participants did an exercise in which they reflected on their past actions and how they could have made a good impression. They also thought about a problematic situation and what they could do to make a good impression. Finally, with the aim of summarizing the intervention to help participants generalize its message better, common daily life situations to obtain good things through one’s actions was discussed (approximately 5 min).

### Intervention Program for the BAA Group

The intervention for the BAA group was also conducted in the gymnasium. The content of the intervention was the same as in the AIM + BAA group, except that AIM was not included. In the BAA group, the intervention was conducted for the same amount of time as that for the AIM + BAA group by prolonging the time spent in exercises and discussions. This intervention was also conducted by the same faculty as that of the AIM + BAA group specializing in cognitive behavioral therapy, with one graduate student as an assistant.

### Measurements

#### Depression

Depression was measured using the Center for Epidemiologic Studies Depression Scale (CES-D) (Radloff, 1977). The CES-D was the primary outcome measure. This study used the Japanese version of the CES-D, as previously validated by Shima et al. (1985). The CES-D consists of 20 items, which participants are asked to rate on a 5-point Likert scale ranging from 0 (rarely or less than 1 day per week) to 3 (almost always that 5 days or more per week). Higher CES-D scores indicate higher depressive tendencies, and a cutoff score of 16 or above is used to identify individuals at risk for clinical depression. This scale has been shown to be reliable and valid for people with depression. In the pre-intervention sample, Cronbach’s alpha was 0.88. The CES-D has been adopted in several surveys of Japanese adolescents (Takakura and Sakihara, 2001). Since it is capable of measuring low levels of depression, it seems appropriate for the purpose of this study.

#### PTSD Symptoms

PTSD symptoms were measured using the Impact of Event Scale-Revised (IES-R) (Weiss and Marmar, 1997). The IES-R was also used as a primary outcome measure. We employed the Japanese version of the IES-R, which was validated by Asukai et al. (2002). The IES-R consists of 22 items, each of which is rated on a 5-point Likert scale ranging from 0 (not at all) to 4 (very). Higher IES-R scores indicate higher PTSD tendencies. A cutoff score of 25 or above is used to help identify individuals at risk for PTSD. The scale has been shown to be reliable and valid for use with survivors of traumatic events. In the pre-intervention sample, Cronbach’s alpha was 0.89.

#### Activation and Avoidance

Activation and avoidance were measured using the Behavioral Activation for Depression Scale-Short Form (BADS-SF) (Manos et al., 1997). In this study, we used the Japanese version of the BADS-SF, as validated by Yamamoto et al. (2015). The BADS-SF consists of 8 items, of which 5 items assess activation and 3 items measure avoidance. Participants rated each item on a 7-point Likert scale ranging from 0 (not at all) to 6 (completely applicable). Higher activation scores indicate higher tendencies for behavioral activation, and higher scores for avoidance indicate higher tendencies for avoidance. This measure has been used previously with Japanese college students. In the pre-intervention sample, Cronbach’s alpha was 0.77 and 0.75 for activation and avoidance, respectively.

#### Resilience

Resilience was measured using the Tachikawa Resilience Scale (TRS) (Nishi et al., 2013). The TRS consists of 10 items and has demonstrated high reliability and validity. Participants rated each item on a 7-point Likert scale ranging from 1 (not at all) to 7 (very true). Higher scores indicate higher levels of resilience. In the pre-intervention sample, Cronbach’s alpha was 0.88.

#### Impression Sheets

An impression sheet created by the first author was used to examine participants’ evaluations of the intervention from multiple perspectives. It consists of six items rated on a 3-point Likert scale and two open-ended questions. Six items were set to measure comprehension, difficulty, efficacy, generalization, confirmation of specific situations, and motivation. An example item is “Did you understand the key point of today’s lesson?” For the open-ended questions, participants were asked to freely write what they thought, felt, and learned in the intervention, and what action they were going to try for behavioral activation.

#### Statistical Analyses

All analyses were performed using SPSS 22 (IBM Japan, Ltd.). Baseline data obtained from the four psychological scales (CES-D, IES-R, BADS-SF, and TRS) were available to all the participants. In total, 239 participants were included in the analyses, excluding 14students who provided incomplete answers to the outcome measures.

The analyses in the current study comprised the following four components: (1) descriptive statistics for all pre- and post-intervention measurements to determine if a parametric analysis was warranted, (2) two-way non-repeated ANOVA to examine the effect of gender distribution on CES-D and IES-R scores pre-intervention, (3) linear mixed modeling (LMM) to examine the intervention effects according to the results of the psychological scales, and (4) a Mann-Whitney *U-*test to examine the intervention effect based on responses provided in the impression sheet.

Descriptive statistics were first calculated for all measurements pre- and post-intervention. Cronbach’s alpha was calculated to assess the internal consistency of the items comprising each scale.

The distribution of males and females across the group indicates that approximately 30% of each group were men. To examine the effect of gender distribution on CES-D and IES-R scores at pre-intervention, a two-way non-repeated ANOVA was used to analyze the effects of gender (male–female) and time (pre–post) on CES-D and IES-R as dependent variables. For the CES-D and IES-R at base line, a two-way repeated ANOVA was used because the within-class correlation coefficient between groups was less than 0.1.

LMM is an analysis that is applied to clustered data. In the case of clustered data, applying ANOVA reduces the power of the test, so applying LMM is more appropriate. For the primary outcome in terms of CES-D and IES-R scores, ICC between groups were computed for the pre-score, post-score, and pre-difference post-score. For CES-D, the pre-score was ICC = 0.04, the post-score was ICC = 0.10, and the difference score was ICC = 0.11. For IES-R, the pre-score was ICC = 0.08, post score was ICC = 0.24, and difference score was ICC = 0.12. Since the post and difference scores of CES-D and IES-R are above 0.1, the cluster should be considered to compare the groups before and after the intervention. Therefore, it is appropriate to use LMM for the intervention effect in the present study. Incomplete data were processed using the full information maximum likelihood method (Arbuckle et al., 1996); LMMs account for correlations that arise from taking multiple measures on the same person by performing multivariate analyses of repeated measures (Arbuckle et al., 1996; Snijders and Bosker, 2011). Data on intervention effects (CES-D, IES-R, BADS-SF, TRS) were collected before and after the intervention. The LMM was used to control for individual and classroom-level differences when examining intervention effects. Universal, school-based interventions, including this study, are usually conducted in a group format within the classroom. The influence of this shared environment may lead to underestimation or overestimation of intervention effects for each participant. Therefore, we treated participants and class as random effects, and the intervention group (BAA or BAA + AI) and timing of measurement (pre- or post-intervention) as fixed factors.

Finally, a Mann-Whitney *U*-test was used to detect group differences in comprehension, difficulty, efficacy, generalization, confirmation of specific situations, and motivation, as assessed from the impression sheet. A significant group difference indicated the effect of AIM on participants’ understanding of and commitment to the treatment.

## Results

### Sample Characteristics and Descriptive Statistics

Means, standard deviations and Cronbach’s alpha for all measurements are summarized in Table 2. Cronbach’s alpha coefficients suggested good internal consistency for all measurements.

**TABLE 2:** Descriptive statistics and result of Linear mixed model.

Variables	AIM + BAA group (*n* = 145)				BAA group (*n* = 108)				
	Pre		Post		Pre		Post		Cronbach’s alpha
	*M*	*SD*	*M*	*SD*	*M*	*SD*	*M*	*SD*	
Age	15.15	0.46	–	–	15.19	0.45	–	–	
Sex (*n* and% of male)	43.00	(30%)	–	–	30.00	(28%)	–	–	
CES-D	14.94	9.84	14.48	9.20	15.44	10.18	16.43	11.09	0.88
IES-R	3.43	5.56	3.33	7.25	5.43	9.71	4.99	8.59	0.89
BADS									
Activation	11.52	6.03	11.21	6.10	10.70	6.58	11.10	7.04	0.77
Avoidance	4.51	3.64	4.15	3.70	4.59	4.60	4.72	4.31	0.75
TRS	43.84	12.47	45.42	12.46	42.47	13.03	44.22	13.52	0.88

	**Intercept**				**Group**				
			
	**β**	**SE**	**95%Cl**	***p***	**β**	***SE***	**95%Cl**	***p***	

CES-D	16.27	1.02	[14.26, 18.28]	<0.001	–1.31	1.36	[-3.98, 1.36]	0.336	
IES-R	5.30	0.73	[3.87, 6.73]	<0.001	–1.50	0.96	[-3.39, 0.38]	0.118	
BADS—activation	10.76	0.60	[9.57, 11.95]	<0.001	0.68	0.80	[-0.90, 2.25]	0.40	
BADS—avoidance	4.81	0.39	[4.05, 5.58]	<0.001	–0.30	0.52	[-0.41, 1.24]	0.57	
TRS	41.67	1.27	[39.18, 44.17]	<0.001	2.20	1.69	[-1.11, 5.52]	0.192	

	**Time**				**Group × Time**			
			
	**β**	**SE**	**95%Cl**	***p***	**β**	**SE**	**95%Cl**	***p***	

CES-D	0.86	1.46	[-2.01, 3.73]	0.557	–1.00	1.92	[-4.78, 2.78]	0.604	
IES-R	–0.13	1.03	[-2.15, 1.89]	0.901	–0.24	1.35	[-2.90, 2.43]	0.862	
BADS—activation	0.17	0.86	[-1.52, 1.87]	0.84	–0.39	1.14	[-2.62, 1.84]	0.73	
BADS—avoidance	0.19	0.56	[-0.91, 1.28]	0.74	–0.33	0.74	[-1.78, 1.11	0.65	
TRS	2.07	1.80	[-1.47, 5.62]	0.251	–0.17	2.38	[-4.85, 4.52]	0.944	

In the present study, the overall mean value of CES-D at pre time point was 15.19 ± 0.73, 14.94 ± 0.94 in the AIM + BAA group and 15.44 ± 1.12 in the BAA group. The number of patients exceeding the cutoff was 79 out of 192 (41.15%) in the overall group, 47 out of 113 (41.59%) in the AIM + BAA group, and 32 out of 79 (40.51%) in the BAA group.

The overall mean of the IES-R at pre-intervention was 4.43 ± 0.55, 3.43 ± 0.71 in the AIM + BAA group and 5.43 ± 0.85 in the BAA group. The number of patients exceeding the cutoff was 6 out of 192 (3.12%) in the overall group, 2 out of 113 (1.77%) in the AIM + BAA group, and 4 out of 79 (5.06%) in the BAA group.

As a reference, Nakaza et al. (2017) surveyed 205 high school students who were affected by the Great East Japan Earthquake as well as the subjects of this study in 2015 and reported the following: the mean of CES-D was 14.10 ± 10.02 points, and the mean of IES-R was 2.43 ± 5.42 points. Seventy-two out of 205 (35.1%) scored above the cut-off of 16 points in CES-D. In IES-R, 4 out of 205 (1.9%) were above the cut-off of 25 points.

### The Effect of Gender Distribution on Depression and PTSD Symptoms Scores at Pre-intervention

#### Depression

ANOVA did not reveal a significant effect of gender [*F*(1, 188) = 3.370, *p* = 0.068], group [*F*(1, 188) = 2.219, *p* = 0.138], and gender × group interaction [*F*(1, 188) = 0.066, *p* = 0.798].

#### PTSD Symptoms

ANOVA did not reveal a significant effect of gender [*F*(1, 188) = 1.219, *p* = 0.271], group [*F*(1, 188) = 0.228, *p* = 0.634], and gender × group interaction [*F*(1, 188) = 0.205, *p* = 0.651].

#### Intervention Effects Assessed by Psychological Scales

Table 3 shows the LMM results. In both the BAA + AI and BAA groups, the interaction was not significantly different for any measurements, including the CES-D, IES-R, activation and avoidance of BADS-SF, and TRS. Significant group and time effects were not observed in any of the measurements.

**TABLE 3:** Result of Mann-Whitney *U*-test on impression sheet.

		*U*	*p*	*R*
1	Comprehension	5631.00	0.001	−0.24
2	Difficulty	6389.00	0.143	−0.10
3	Efficacy	6240.00	0.071	−0.12
4	Generalization	6059.50	0.023	−0.15
5	Confirmation of specific situations	6765.00	0.532	−0.04
6	Motivation	6026.00	0.025	−0.15

### Intervention Effects Assessed by Impression Sheets

Table 3 presents the results of the Mann-Whitney *U*-test. As shown, comprehension (*U* = 5631.00, *p* < 0.001, *r* = −0.24), generalization (*U* = 6059.50, *p* = 0.023, *r* = −0.15) and motivation (*U* = 6026.00, *p* = 0.025, *r* = −0.15) were higher in the AIM + BAA group, compared to the BAA group.

For the open-ended questions, the answers to “Please write what you want to remember or try from today’s class” in the order of frequency were: “I want to take action that will result in a good outcome for me and others,” “I want to play a game of catch with other friends and family,” and “I’d like to examine what other kinds of games there are in AIM.” The answers to the question “Please write anything you feel about today’s class” in the order of frequency were: “I enjoyed the game and would do it again,” “It was more fun than the usual class because we could play games,” and “Many of the lessons were applicable to me.”

## Discussion

The purpose of this study was to evaluate the effects of BAA and AIM on the primary outcomes of symptoms of depression and PTSD and secondary outcomes of behavioral activation, avoidance, and resilience. This study also examined adolescents’ motivation to participate in an intervention. The findings suggested that BAA did not improve the primary and secondary outcomes; however, the results of the impression sheet indicated that the AIM + BAA group reported higher comprehension, generalization, and motivation after the intervention. In the current study, there was no difference in depression and PTSD symptoms scores due to differences in gender distribution. It was considered unlikely that gender factors would affect the results of the current study.

The results of this study did not confirm the effectiveness of BAA in reducing symptoms of depression and PTSD. This result was inconsistent with an earlier report by McCauley et al. (2016), which showed significant symptom reduction in depression and PTSD after a 14-session BAA program. Although most BAA programs for depression include 4-20 sessions (McCauley et al., 2016), the current BAA program included only one session, which may have been insufficient for reducing symptoms of depression and PTSD.

Regarding treatment motivation, participants in the AIM + BAA group reported higher motivation compared to the BAA group. This suggested that one AIM session may contribute to improved treatment motivation. AIM creates a safe environment and encourages engagement and participation in interventions. For example, for the open-ended questions on the impression sheet, some participants wrote responses such as “I enjoyed communicating,” “I think everyone enjoyed it,” “We got to know each other,” and “It was fun to cooperate with everyone,” suggesting they perceived a safe environment during the intervention. This safe environment may have also encouraged comprehension and generalization of the intervention components. There were no negative statements that indicated issues such as resistance or refusal. Westra (2004) noted that higher motivation in the pre-treatment period or early stages of treatment predicts higher treatment involvement and lower dropout rates. Motivational procedures, such as AIM, in the early stages of treatment may contribute to improving the quality of adolescent treatment and school-based interventions.

Comprehension and generalization as assessed by the impression sheet also showed significant differences in the AIM + BAA group compared to the BAA group. It is possible that the combination of applied improvisation and another intervention procedure increased the motivation for the intervention and promoted understanding of the program. Under generalization, there was a comment on the impression sheet: “I am glad I took the plunge and participated.” It is possible that the participants experienced and understood that behavioral activation can lead to positive reinforcements which brings about enjoyable feelings.

The current study has some methodological limitations. First, because all participants in both groups attended the same school, they could have discussed the intervention component, which may have caused treatment contamination problems. Contamination can lead to an underestimation of the intervention effect. To obtain unbiased and valid data, a more rigorous research design, such as a cluster randomized trial with participants from several different schools, should be employed. Second, while the impression sheets showed significant differences between the groups on several items (comprehension, generalization, and motivation), the effect size (*r*-value) was small, which indicates that a single session has a limited effect on intervention. However, the purpose of this study was to examine the effect of using AIM on treatment motivation for the intervention. Although the effect size was small, treatment motivation was higher in the AIM + BAA group than in the BAA group, suggesting that it is possible to improve motivation even in a single session. Third, as motivation was assessed using one item on an impression sheet, the current results may have been influenced by measurement bias and therefore should be re-examined using reliable and valid measures. Fourth, all measurements in this study consisted of self-report scales. Future studies should use multiple measurement methods, such as parent-report, clinician-report, observations, or interviews, to allow for more reliable and objective measurements. Fifth, this study did not identify any other factors that may have influenced the development of symptoms. The subjects in this study were also not compared to other similar groups. The possibility that other factors associated with the subjects of this study may have influenced the results cannot be denied. However, this study is significant in that it empirically examines the specific steps that can be taken to increase the effectiveness of BAA among Japanese high school students.

## Conclusion

In conclusion, this study showed that AIM enhanced adolescents’ motivation for and commitment to an intervention. Treatment motivation is an important issue in adolescent therapy, and AIM may help to solve this problem. In the future, further research is needed to create more sophisticated psychological interventions for adolescents following large-scale disasters. Further implementation of the intervention program combined with applied improvisation is expected to promote intervention effects even in children with high anxiety and developmental disabilities.

## Data Availability Statement

The raw data supporting the conclusions of this article will be made available by the authors, without undue reservation.

## Ethics Statement

The studies involving human participants were reviewed and approved by the Ethics Committee of J. F. Oberlin University. Written informed consent to participate in this study was provided by the participants’ legal guardian/next of kin.

## Author Contributions

CS contributed to the conception and design of this study, the statistical analysis, and the writing of the first draft of the manuscript. SK contributed to the conception and design of this study, as well as the communication and coordination with the intervention school. YN contributed to the organization of the database and to the writing of part of the manuscript. DI contributed to communication and coordination with the intervention school and to writing part of the manuscript. FT contributed to the statistical analysis and to the writing of part of the manuscript. RI contributed key ideas in the conception and design of this study. All authors contributed to the article and approved the submitted version.

## Conflict of Interest

The authors declare that the research was conducted in the absence of any commercial or financial relationships that could be construed as a potential conflict of interest.

## Publisher’s Note

All claims expressed in this article are solely those of the authors and do not necessarily represent those of their affiliated organizations, or those of the publisher, the editors and the reviewers. Any product that may be evaluated in this article, or claim that may be made by its manufacturer, is not guaranteed or endorsed by the publisher.
