# Supplementary material for: Applied Improvisation Enhances the Effects of Behavioral Activation on Symptoms of Depression and PTSD in High School Students Affected by the Great East Japan Earthquake
Source: Front Psychol. 2021 Aug 26;12:687906. doi: 10.3389/fpsyg.2021.687906 (PMC8426506; doi:10.3389/fpsyg.2021.687906)
Supplement: Supplementary file 1 [file Table_1.DOCX]

| **S1 Table. Impression sheet (6 items).** | | | |
| --- | --- | --- | --- |
|  | | | |
| No. | Intention | Item | Evaluation |
| 1 | Comprehension | Did you understand the key point of today's lesson? | 1 (I didn't understand) 2 (I understand) 3 (I understand very well) |
| 2 | Difficulty | Did you find today's lesson difficult? | 1 (Difficult) 2 (Just right) 3 (Easy) |
| 3 | Efficacy | Did you find today's lesson useful for you? | 1 (It doesn't help much) 2 (It seems a little useful) 3 (Very useful) |
| 4 | Generalization | Did you find the lesson practical for everyday use? | 1 (Can't actually use) 2 (A little usable) 3 (Actually usable) |
| 5 | Confirmation of specific situations | Did you identify a situation in daily life where you can actually use what you learned today? | 1 (No idea) 2 (One comes to mind) 3 (Some come to mind) |
| 6 | Motivation | Would you want to participate in a similar lesson again? | 1 (I do not want to participate) 2 (I may try participating) 3 (I want to participate again) |
| 2 open-ended questions | | | |
| 1 | What do you remember from today’s class? | | |
| 2 | What are your impressions of today's class? | | |
